# Supplementary material for: Frailty Severity and Cognitive Impairment Associated with Dietary Diversity in Older Adults in Taiwan
Source: Nutrients. 2021 Jan 28;13(2):418. doi: 10.3390/nu13020418 (PMC7911853; doi:10.3390/nu13020418)
Supplement: Supplementary file 1 [file nutrients-13-00418-s001.pdf]

Table S1. Comparison of selected food intake frequency (times/weekly) by dietary diversity score in older adults in the 2014–2016 Nutrition and Health Survey in Taiwan.

Characteristic	DDS				*p* for trend
	≤ 3	4	5	6	
***n* (%)**	76 (6.0)	287 (23.7)	532 (49.7)	219 (20.6)	
Dairy products	0.15±0.04	0.30±0.04	0.32±0.03	1.00±0.09	<0.001
Whole grains	0.14±0.06	0.33±0.06	0.46±0.05	0.52±0.07	<0.001
Vegetables	2.17±0.24	2.70±0.13	3.38±0.10	3.83±0.25	<0.001
Mushroom	0.98±0.23	0.98±0.11	1.82±0.15	2.10±0.34	<0.001
Pickled vegetables	0.13±0.05	0.15±0.03	0.12±0.01	0.08±0.01	0.074
Fruits	0.86±0.12	0.75±0.06	1.36±0.05	1.69±0.09	<0.001
Soybean products	0.55±0.14	0.43±0.04	0.47±0.03	0.47±0.04	0.967
Fish and seafood	0.98±0.12	0.84±0.06	1.12±0.07	1.28±0.11	0.001
Eggs	0.37±0.05	0.35±0.03	0.41±0.03	0.39±0.02	0.348
Livestock	0.06±0.04	0.01±0.00	0.02±0.00	0.02±0.00	0.291
Poultry	0.21±0.04	0.18±0.02	0.20±0.01	0.20±0.02	0.626
Processed meat	0.15±0.05	0.11±0.02	0.13±0.01	0.14±0.03	0.767
Nuts and seeds	0.09±0.02	0.20±0.03	0.30±0.03	0.41±0.05	<0.001
Tea	0.32±0.07	0.34±0.04	0.41±0.04	0.40±0.05	0.201
Coffee	0.36±0.12	0.21±0.05	0.27±0.03	0.28±0.04	0.816
Snacks	0.32±0.04	0.38±0.06	0.39±0.03	0.57±0.06	0.003
Fried food	0.03±0.01	0.02±0.01	0.03±0.01	0.01±0.00	0.515
Sweetened beverage	3.98±0.83	2.48±0.43	2.28±0.20	2.34±0.39	0.145

P for trend were performed by linear regression.
